# Beyond the operating room: do hospital characteristics have an impact on surgical site infections after colorectal surgery? A systematic review

**DOI:** 10.1186/s13756-021-01007-5

**Published:** 2021-09-30

**Authors:** Rui Malheiro, Bárbara Peleteiro, Sofia Correia

**Affiliations:** 1 EPIUnit—Instituto de Saúde Pública, Universidade do Porto, Rua das Taipas 135, 4050-091 Porto, Portugal; 2 Department of Public Health and Forensic Sciences and Medical Education, Faculdade de Medicina, Universidade do Porto (University of Porto Medical School), Porto, Portugal; 3 Laboratory for Integrative and Translational Research in Population Health (ITR), Porto, Portugal

**Keywords:** Characteristics, Colorectal surgery, Facilities, Hospital, Review, Surgical site infection, Surveillance

## Abstract

**Background:**

Hospital characteristics have been recognized as potential risk factors for surgical site infection for over 20 years. However, most research has focused on patient and procedural risk factors. Understanding how structural and process variables influence infection is vital to identify targets for effective interventions and to optimize healthcare services. The aim of this study was to systematically review the association between hospital characteristics and surgical site infection in colorectal surgery.

**Main body:**

A systematic literature search was conducted using PubMed, Scopus and Web of Science databases until the 31st of May, 2021. The search strategy followed the Participants, Exposure/Intervention, Comparison, Outcomes and Study design. The primary outcome of interest was surgical site infection rate after colorectal surgery. Studies were grouped into nine risk factor typologies: hospital size, ownership affiliation, being an oncological hospital, safety-net burden, hospital volume, surgeon caseload, discharge destination and time since implementation of surveillance. The STROBE statement was used for evaluating the methodological quality.

A total of 4703 records were identified, of which 172 were reviewed and 16 were included. Studies were published between 2008 and 2021, and referred to data collected between 1996 and 2016. Surgical site infection incidence ranged from 3.2 to 27.6%. Two out of five studies evaluating hospital size adjusted the analysis to patient and procedure-related risk factors, and showed that larger hospitals were either positively associated or had no association with SSI. Public hospitals did not present significantly different infection rates than private or non-profit ones. Medical school affiliation and higher safety-net burden were associated with higher surgical site infection (crude estimates), while oncological hospitals were associated with higher incidence independently of other variables. Hospital caseload showed mixed results, while surgeon caseload and surveillance time since implementation appear to be associated with fewer infections.

**Conclusions:**

Although there are few studies addressing hospital-level factors on surgical site infection, surgeon experience and the implementation of a surveillance system appear to be associated with better outcomes. For hospitals and services to be efficiently optimized, more studies addressing these variables are needed that take into account the confounding effect of patient case mix.

## Background

Surgical site infection (SSI) is the third most common healthcare-associated infection (HAI) [1], and it is known to have a high impact on hospital length of stay, expenditure, surgical morbidity and mortality [1–3]. According to the latest report from the European Centre for Disease Prevention and Control, open colon surgery was the procedure associated with the highest risk of SSI (10.1 per 100 operations) followed by laparoscopic colon surgery (6.4 per 100 operations) [4]. Given its burden, efforts have been made to identify modifiable risk factors. In their guideline for the prevention of SSI in 1999, the Centers for Disease Control and Prevention (CDC) acknowledged that the risk of SSI is influenced by the characteristics of the patient, procedure, personnel and hospital [5]. Based on the same rationale, the National Healthcare Safety Network (NHSN), the North American surveillance system for HAI, combine facility, patient and procedure-level variables in their SSI risk adjustment models to predict the number of expected infections [6]. However, most research has focused on patient and procedural risk factors. Similarly, preventive interventions—either isolated or in a bundle—have focused exclusively on optimizing patient condition and delivering the surgical procedure as safely as possible [7–10]. Hospital characteristics have been consistently overlooked. Even though most may be deemed as non-modifiable, they are proxy indicators of unmeasured variables, such as cleanliness, structural and organizational characteristics, staffing or training [11], all of which may be potential targets for improvement. Better structural resources and better processes should provide better outcomes. Thus, understanding how structural and process variables may influence SSI is vital to pinpoint effective interventions and to optimize healthcare services.

The aim of this study was to systematically review the published literature regarding the association between hospitals’ characteristics, including services provided, and SSI incidence after colorectal surgery.

## Methods

### Search strategy

The search strategy followed the Participants, Exposure/Intervention, Comparison, Outcomes and Study (PE/ICOS) design [12]. PubMed, Scopus and Web of Science databases were searched, with no date limit, using the following query: (colorectal OR colon OR rectal) AND (surgical site infection OR wound infection OR skin infection) AND (effect OR risk OR association OR impact OR relation* OR influence OR outcome). All sources were last searched on May 31st, 2021, and backward citation tracking was conducted for all included articles. This systematic review was undertaken using the PRISMA (Preferred Reporting Items for Systematic Reviews and Meta-analyses) guidelines [13] but was not registered in the PROSPERO database.

### Inclusion and exclusion criteria

The inclusion criteria were as follows: (1) articles written in English, Portuguese, Spanish, Italian, French or German, (2) not a review article, editorial, comment, guideline or descriptive study, (3) patients submitted to colorectal surgery, (4) analysis of risk factors representing hospital characteristics, services or organization (no patient or procedure-related risk factors), (5) SSI as an outcome and (6) studies with odds ratio (OR) or relative risk (RR), or raw data allowing the estimation of those measures of association.

### Data extraction

RM and BP independently reviewed titles and abstracts of all records retrieved from electronic searches, applying the aforementioned criteria. Any disagreements were solved through a consensus discussion, or involving SC. Full texts and supplement material (when available) of all identified studies were then reviewed by the same researchers. Given that all included studies were observational, the STROBE checklist was used for evaluating their methodological quality [14]. This is a checklist of 22 items that should be included in reports of observational studies. Each sub-item was graded as 1, if the study reported them as recommended; 0, if the sub-item was missing from the study; or 0.5, if the sub-item was included but only partially met the recommendation. As some sub-items could be non-applicable, the maximum score ranged from 24 to 30.

Data on first author, publication year, language, study design, country, recruitment period, surgical procedures considered, procedure codes used, databases used, type and criteria of SSI and study size were retrieved. Missing data was registered as such, and no assumptions were made. Nonetheless, authors were contacted to retrieve necessary data when studies fulfilling the inclusion criteria had missing data. When applicable, information on whether the hospital had an infection control team and whether surveillance included post-discharge diagnosis were also retrieved.

The primary outcome of interest was SSI rate after colorectal surgery, whether superficial incisional, deep incisional or organ/space, as defined by the CDC [10]. Measures of association and their respective 95% confidence interval (CI) were retrieved, or estimated when raw data was available.

Studies were grouped in nine risk factor typologies: hospital size, for those studies that estimated the association between hospital’s number of beds and SSI; hospital ownership, when the analysis focused on whether hospitals were public, private or non-profit; medical school affiliation, for the comparison of teaching versus non-teaching hospitals; Oncological hospitals, for studies researching whether a hospital being a specialized oncological center had an impact on SSI incidence; safety-net burden, defined as the proportion of patients a hospital treats who are either uninsured or insured by Medicaid, an American state program that helps with healthcare costs for people with limited income and resources; hospital volume of procedures, when the risk factor analyzed was the number of colorectal procedures performed at each hospital; surgeon volume of procedures, when the risk factor was the number of colorectal procedures performed per surgeon, rather than per hospital; post-discharge destination, analyzing if patients discharged to their homes had different outcomes when compared to those discharged to skilled facilities; and surveillance time, for studies estimating the impact of surveillance programs over the years on SSI rates.

## Results

**Fig. 1:**
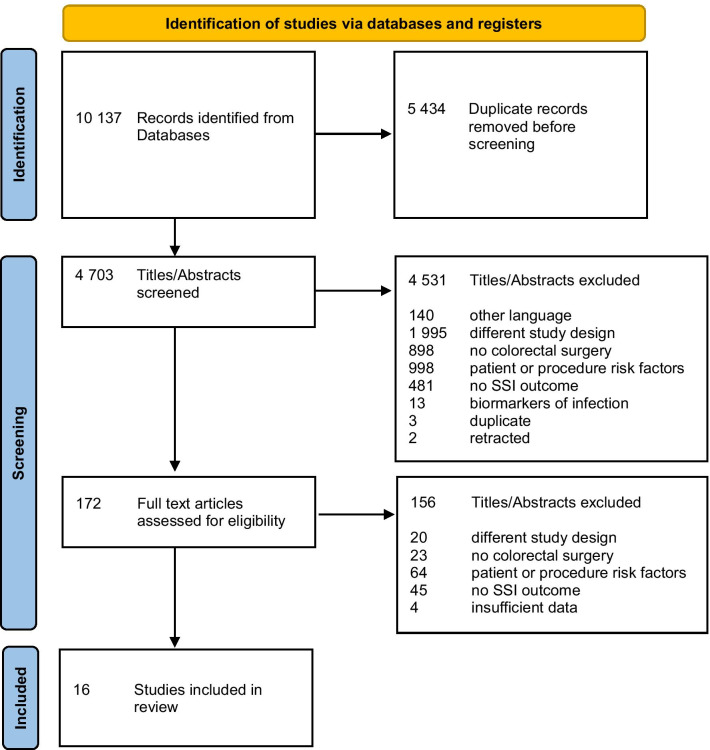
Flow diagram of the study selection process

A total of 4703 records were identified through the databases’ search, after duplicates were removed, of which the full text of 172 was reviewed, and 16 were included in our systematic review (Fig. 1). No additional article was included following backward citation tracking. Six studies were from the United States (U. S.), two from Italy, two from Spain, one each from Australia, China, Germany, the Netherlands and Switzerland, and one was an international study conducted across Australia, Singapore, South Korea and 12 European countries. Apart from the Dutch study, published in 2008, the remaining 15 were published in the last decade, between 2011 and 2021, with data collected between 1996 and 2016. The 16 studies included comprised 1,314,608 colorectal procedures, and are described in detail in Table 1. SSI incidence ranged from 3.2 to 27.6%, and the methodological quality score varied between 11 and 25.

**Table 1:** Characteristics of all included studies

First author, year, country	Description	Study design and PICO	Study results
Abbas 2019 [26] Multiple countries English	Large-scale international study to determine the time-trend of surgical site infection (SSI) incidence in SSI surveillance networks. Networks identified through systematic literature review were provided with standardized data template	Cohort *Population* Colorectal surgery (no source) *Intervention* Surveillance over time 1) One-year increase in surveillance time 2) X years of surveillance *Comparison* 1) Year One in surveillance time 2) X-1 years of surveillance *Outcome* SSI rate (no source) *STROBE Score* 25 in 29	*Study size*: 320 123 10 networks included 8.5 (IQR 7–11) median years of surveillance *Relative Risk* Intervention 1) 1) 2 years 0.92 (0.89–0.96) 2) 3 years 0.90 (0.87–0.94) 3) 4 years 0.91 (0.87–0.95) 4) 5 years 0.86 (0.83–0.90) 5) 6 years 0.92 (0.87–0.96) 6) 7 years 0.84 (0.79–0.89) 7) 8 years 0.86 (0.80–0.92) Intervention 2) 1) X = 3 - 0.98 (0.94–1.02) 2) X = 4 − 1.00 (0.96–1.05) 3) X = 5–0.95 (0.91–0.99) 4) X = 6–1.06 (1.01–1.12) 5) X = 7–0.92 (0.86–0.98) 6) X = 8–1.02 (0.04–1.11)
Angel García 2020 [17] Spain English	Study data from nine public hospitals in Murcia, Spain, from January 2006 to December 2015. The study developed two risk-adjustment models based on multiple logistic regression, without considering hospital bed size as a candidate variable	Cohort *Population* Colorectal surgery (ICD-9) *Exposure* 1) Bed size > 500 2) Bed size 250–500 *Comparison* Bed size < 250 *Outcome* SSI (ICD-9 codes) *STROBE score* 17 in 24	*Study size* 6 325 423 SSIs (7.32%) *Odds ratio (OR) (overall SSI, univariate analysis)* 1) 0.54 (0,41–0,72) 2) 0.95 (0.74–1.23)
Du 2019 [16] China English	Multicenter surveillance of radical resection of colon and rectal carcinoma in 26 tertiary hospitals in 14 cities, from January 2015 to June 2016 Surveillance made by infection control professionals until discharge, using a real-time nosocomial infection surveillance system, and by telephone 30 days postoperatively	Cohort *Population* Radical resection of colon and rectal carcinomas (ICD-9) *Exposure* Beds < 2500 *Comparison* Beds ≥ 2500 *Outcome* SSI (CDC 1992) *STROBE score* 11 in 30	*Study size* 5 729 3 406 radical resection of colon carcinoma 2 323 radical resection of rectal carcinoma 206 SSIs (3.60%) 87 SSIs after colon resection (2.55%) • 32 superficial (0.94%) • 19 deep (0.56%) • 36 organ space (1.06%) 119 SSIs after rectal resection (5.12%) • 53 superficial (2.28%) • 26 deep (1.11%) • 40 organ space (1.72%) *OR (Multivariable analysis for overall SSI)* 0.644 (0.451–0.921) resection of colon carcinoma 0.513 (0.356–0.739) resection of rectal carcinoma
El Aziz 2020 [25] United States English	Six-year longitudinal study using the American College of Surgeons—National Surgical Quality Improvement Program (ACS-NSQIP) database, an American quality improvement program gathering abstract patient information through predesigned data extraction sheets manage by trained data abstractors from all participating institutions SSIs assessed in-hospital before discharge and after discharge until post-operative day 30 ORs adjusted for age > 80 years old, gender, race, body mass index, diabetes mellitus, current smoker within one year, dyspnea, functional health status prior to surgery, history of severe chronic obstructive pulmonary disease, ascites, congestive heart failure in 30 days before surgery, hypertension requiring medication, dialysis, disseminated cancer, open wound infection, steroid use for chronic condition, > 10% loss bodyweight in the last six months, bleeding disorders, transfusion > 1 unit red blood cells 72 h before surgery, pre-operative albumin and hematocrit, diagnosis, extent of resection, operative approach, diversion, operation time, any surgical complication before discharge, any medical complication before discharge, days from operation to discharge	Cohort *Population* Elective surgery for colon or rectal cancer, using Current Procedural Terminology (CPT) codes *Exposure* 1) Discharge to skilled facility 2) Discharge to rehabilitation center 3) Discharge to separate acute care *Comparison* Discharged home *Outcome* SSI (ACS-NSQIP 2016) *STROBE score* 21 in 27	*Study size* 108 617 3476 total SSIs (3.2%) 1396 superficial SSIs (1.3%) 349 Deep SSIs (0.3%) 1915 organ space SSIs (1.8%) *ORs (overall SSIs, adjusted)* 1) 1.02 (0.87–1.20) 2) 1.03 (0.81–1.31) 3) 1.25 (0.74–2.09)
Furuya-Kanamori 2017 [20] Australia English	New South Wales Admitted Patient Data Collection contains data on all admitted patient services provided by public and private hospitals in the region Subset population of adult patients who underwent colorectal surgery between January 2002 and December 2013 The annual volume of colorectal surgery in public hospitals was categorized into tertiles, per surgical procedure: low-volume hospitals performed < 45procedures/year, mid-volume performed 45–115 procedures/year and high-volume performed > 115 procedures/year Outcome includes in-hospital infection only	Cohort *Population* Colorectal surgery (ICD-10, Australian Modification) *Exposure* 1) Mid-Hospital Volume 2) High-Hospital Volume *Comparison* Low-Hospital Volume *Outcome* Surgical site infection (ICD-10) *STROBE score* 20.5 in 28	*Study size* 58 096 cases from 59 hospitals Incidence: 9.64% (9.40–9.88%) *OR (overall SSI, crude analysis)* 1) 1.23 (1.08–1.40) 2) 1.50 (1.34–1.69) When risk-adjusted SSI rates per 1000 admissions were examined, low-volume hospitals performed better for colorectal procedures (91.7 for low, 96.7 for mid and 96.7 for high-volume public hospitals)
Manilich 2013 [23] United States English	Single-center study, with exclusion of outpatient surgical cases. 30-day follow-up by letter or phone call Surgeon volume determined by the number of procedures in each major category that a surgeon performed in 2 years—colorectal surgical procedure as the unit of analysis	Cohort *Population* Major abdominal or transanal colorectal surgery (no source) *Exposure* Surgeon volume < 20 procedures *Comparison* Surgeon volume ≥ 20 procedures *Outcome* SSI (ACS-NSQIP) *STROBE score* 23 in 29	*Study size* 3 552 cases by 15 surgeons 300 incisional SSIs (8.4%) *OR (overall SSI, adjusted OR)* 1.38 (1.06–1.79)
Mannien 2008 [28] Netherlands English	Data from 1996 to 2006 from the Dutch National Nosocomial Surveillance Network. Hospital participation is voluntary. Hospitals can annually decide which surgical procedures to include, and post-discharge surveillance is strongly recommended OR adjusted for post-discharge surveillance, American Society of Anesthesiologists (ASA) classification, wound contamination class, duration of surgery, duration of preoperative hospitalization and emergency procedure	Cohort *Population* Colectomy (no source) *Intervention* Surveillance 1) 1-year increase in surveillance time to operation *Comparison* 1-less year in surveillance time to operation *Outcome* SSI (CDC 1992) *STROBE score* 18.5 in 27	*Study size* 3 031 370 SSIs (12.2%) *OR (overall SSI, adjusted)* 1) 0.92 (0.83–1.02)
Merkow 2013 [19] United States English	Multicenter study using centers participating in the ACS-NSQIP, that collects comprehensive data from > 500 hospitals, including 51 National Cancer Institutes Age, race, ASA class, functional status, preoperative albumin level, hypertension, chronic obstructive pulmonary disease, chemoradiation, disseminated cancer and case complexity were all significantly different at baseline. Adjustment for differences in patient demographics and risk factors, as well as surgical complexity	Cohort *Population* Colorectal cancer surgery (CPT) *Exposure* Oncological Hospital 1) National Cancer Institute *Comparison* Non-oncological hospital *Outcome* SSI (ACS NSQIP) *STROBE score* 18.5 in 28	*Study size* 52,265 from 310 hospitals Incidence 7.7% superficial SSIs 4.8% deep or organ/space SSIs *OR (adjusted)* Superficial: 1.35 (1.08–1.70) Deep or organ/space: 1.17 (0.98–1.40)
Schröder 2018 [18] Germany English	Data from surgical site infection module of the German national nosocomial infection surveillance system, the component for surgical site infections, which is patient based and voluntary. SSI following laparoscopic colon resection from 145 hospitals (44 public, 65 non-profit and 36 private) and following open colon resection in 159 (45, 67 and 37, respectively)	Cohort *Population* Colon procedures (national codes) *Exposure* Public ownership Non-profit ownership Bed size < 400 *Comparison* Private ownership Bed size < 400 *Outcome* SSI *STROBE score* 19 in 29	*Study size* 28,291 • 19 453 open colon procedures • 8838 laparoscopic colon procedures *ORs (overall SSIs, multivariate analysis)* 1.12 (0.86–1.47) for public ownership 0.85 (0.66–1.09) for non-profit ownership 0.81 (0.51–1.29) for bed size < 400
Serra-Aracil 2011 [15] Spain English	Multicenter study of 19 public hospitals in Catalonia, Spain, between June 2007 and March 2008 Colon operation defined as any resection above the peritoneal reflection. Rectal operation defined as any resection below the same point. Inclusion criteria were the application of all preventive measures and rectal cancer operations with oncologic resections. Outpatient visit after 30 days	Cohort *Population* Elective operations for colon or rectal cancer *Exposure* 1) > 500 beds 2) 250–500 beds *Comparison* < 250 beds *Outcome* SSI (CDC 1992) *STROBE score* 18 in 29	Study Size: 611 383 colon cancer operation 89 total SSIs (23.2%) • 49 superficial SSIs (12.8%) • 8 deep SSIs (2.1%) • 32 organ space SSIs (8.4%) 228 rectal cancer operation 63 SSIs (27.6%) • 31 superficial SSIs (13.6%) • 13 deep SSIs (5.7%) • 19 organ space SSIs (8.3%) *OR (univariate analysis)* Colon cancer operations Overall SSI 1) 0.48 (0.25–0.95) 2) 0.41 (0.17–0.95) Incisional SSI 1) 0.36 (0.18–0.76) 2) 0.26 (0.09–0.68) Organ/space SSI 1) 1.25 (0.41–5.68) 2) 1.52 (0.39–7.80) Rectal cancer operations Overall SSI 1) 0.69 (0.30–1.67) 2) 0.68 (0.24–1.94) Incisional SSI 1) 0.89 (0.35–2.63) 2) 0.90 (0.27–3.13) Organ/space SSI 1) 0.51 (0.16-2.00) 2) 0.49 (0.08–2.54)
Spolverato 2019 [21] Italy English	Data from National Italian Hospital Discharge Dataset, from January 2002 to November 2014 Adult patients only Hospital volume calculated as the average annual number of rectal cancer procedures performed at each hospital, during study period, divided into tertiles (respectively 1–12, 13–31, > 31 surgeries/year) Main outcome is failure to rescue, defined as the mortality rate among patients with complications, which is why there is no adjusted analysis specifically for SSI; however, low-volume hospitals, in multivariable analysis, are associated with higher rate of failure to rescue and any complication, when compared with high volume hospitals	Cohort *Population* Major surgical procedure for primary rectal cancer (ICD-9) *Exposure* 1) High-volume hospital 2) Intermediate-volume hospital *Comparison* Low-volume hospital *Outcome* SSI (ICD-9) *STROBE score* 20.5 in 30	*Study size* 75 280 3.9% SSI incidence *OR (overall SSI, crude analysis)* 1) 0.99 (0.90–1.08) 2) 0.95 (0.87–1.04)
Staszewicz 2014 [29] Switzerland English	Data collected from 1998 to 2010 from the Swissnoso consortium, a voluntary participation surveillance network of Swiss public hospitals OR adjusted for age, sex, ASA Score ≥ 3, delay from admission to operation > 2 days, emergency, antibiotic prophylaxis, contamination class ≥ 3, multiple procedures, laparoscopy, duration > T, re-intervention for non-infectious complications	Cohort *Population* Colectomy (no source) *Intervention* Surveillance 1) time to operation *Outcome* SSI (CDC 1992) *STROBE score* 18 in 28	*Study size* 7411 1349 SSIs (18.2%) 555 superficial SSIs (7.5%) 308 deep SSIs (4.2%) 486 organ/space SSIs (6.6%) *OR (overall SSI, adjusted)* 1.05 (1.03–1.07)
Tserenpuntsag 2014 [11] United States English	Multicenter study of 174 New York State hospitals, with mandatory surveillance of SSIs following colon procedures through the NHSN, including post-discharge detection of SSI. Authors used 2009–2010 data of an audit of the surveillance program, performed by trained program staff certified in infection control If a small bowel procedure, kidney transplant, liver transplant, or bile duct, liver pancreatic or rectal procedure was performed through the same incision, and it was not clear which procedure was associated with infection, the SSI was attributed to 1 of the above listed procedures	Case-control *Population* Colon procedures, using ICD-9 codes *Exposures* Teaching hospitals Bed size > 500 *Comparison* Nonteaching hospitals Bed size ≤ 500 *Outcome*: SSI (CDC 1992) *STROBE Score*: 18.5 in 28	*Study Size*: 2 656 cases from 175 hospitals 698 SSIs identified • 355 superficial incisional • 343 deep incisional and organ space *ORs (overall SSI, bivariable analysis)*: Teaching hospitals: 1.88 (1.55–2.95) for superficial 1.86 (1.45–2.39) for deep incisional and organ space Bed size: 2.32 (1.82–2.95) for superficial incisional and 2.08 (1.54–2.80) for deep and organ space
Vicentini 2019 [27] Italy English	32 Piedmont hospitals (primary, secondary and tertiary) participating in the voluntary Italian surveillance system for SSIs, using data from January 2009 to December 2015 Surveillance must be performed at least 6 months/year or a minimum of 50 procedures must be monitored Surveillance time is equivalent to the number of years of participation in a surveillance program	Cohort *Population* Colon surgery (ICD-9) *Intervention* Surveillance 1) Participating in surveillance program for over 5 years 2) 1-unit increase in the number of monitored procedures 3) 1-year increase in surveillance time *Comparison* 1) No participation in surveillance network *Outcome* SSI (ECDC) *STROBE score* 16 in 27	*Study size* 6 060 595 SSIs (9.83%), 172 post-discharge 370 incisional SSIs (6.13%) 96 deep SSIs (1.59%) 97 organ/space SSI (1.60%) *RR (overall SSI)* 1) 0.64 (0.46–0.90) 2) 0.99 (0.98-1.00) 3) 0.93 (0.89–0.97)
Wang 2021 [22] United States English	Safety-net hospitals are mandated to treat patients regardless of their ability to pay, and consequently carry a high safety-net burden (SNB), defined as the proportion of patients who are either uninsured or Medicaid-insured. Hospitals were stratified into tertiles of low, medium and high SNB Data from State Inpatient Databases (2009–2014) Hospital volume stratified into quartiles by colectomy case volume Adult patients only	Cohort *Population* Colectomy (ICD-9) *Exposure* 1. High safety-net burden 2. Medium safety-net burden 3. Hospital volume—4th quartile 4. Hospital volume—3rd quartile 5. Hospital volume—2nd quartile *Comparison* 1. Low safety-net burden (1–2) 2. Hospital volume—1st quartile (3–5) *Outcome* SSI (ICD-9) *STROBE score* 21.5 in 30	*Study size* 459 568 29 117 SSIs (6.3%) *OR (overall SSI, crude analysis)* 1. 1.35 (1.31–1.40) 2. 0.97 (0.94-1.00) 3. 0.51 (0.50–0.53) 4. 0.64 (0.62–0.66) 5. 0.70 (0.68–0.73)
Yi 2018 [24] United States English	Administrative databases used for colorectal surgical patients discharged in years 2013 and 2014, in a hybrid tertiary referral central with 8 campuses and 2247 beds. Over 80% of study patients were from campuses with high colorectal surgery volume Volume of surgery determined by the actual number of patients operated on per surgeon. High volume surgeon with case volume of more than 34 cases in the last 2 years, medium volume with case volume between 14 and 34, and low volume with fewer than 14 cases	Cohort study *Population* Colorectal procedures (ICD-9) *Exposure* Medium-volume Surgeon Low-volume Surgeon *Comparison* High-volume Surgeon *Outcome* SSI (no source provided) *STROBE Score* 16,5 in 27	*Study size* 1 190 cases by 44 surgeons *ORs (overall SSI, adjusted)* 0.23 (0.08–0.65) for medium-volume surgeons 0.39 (0.09–1.71) for low-volume surgeons

ACS-NSQIP, American College of Surgeons-National Surgical Quality Improvement Program; ASA, American Society of Anesthesiologists; CDC, Centers for Disease Control and Prevention; CPT, Current Procedural Terminology; ECDC, European Centre for Disease Prevention and Control; ICD, International Statistical Classification of Diseases and Related Health Problems; IQR, interquartile range; NHSN, National Healthcare Safety Network; OR, odds ratio; SNB, safety-net burden; SSI, surgical site infection

**Fig. 2:**
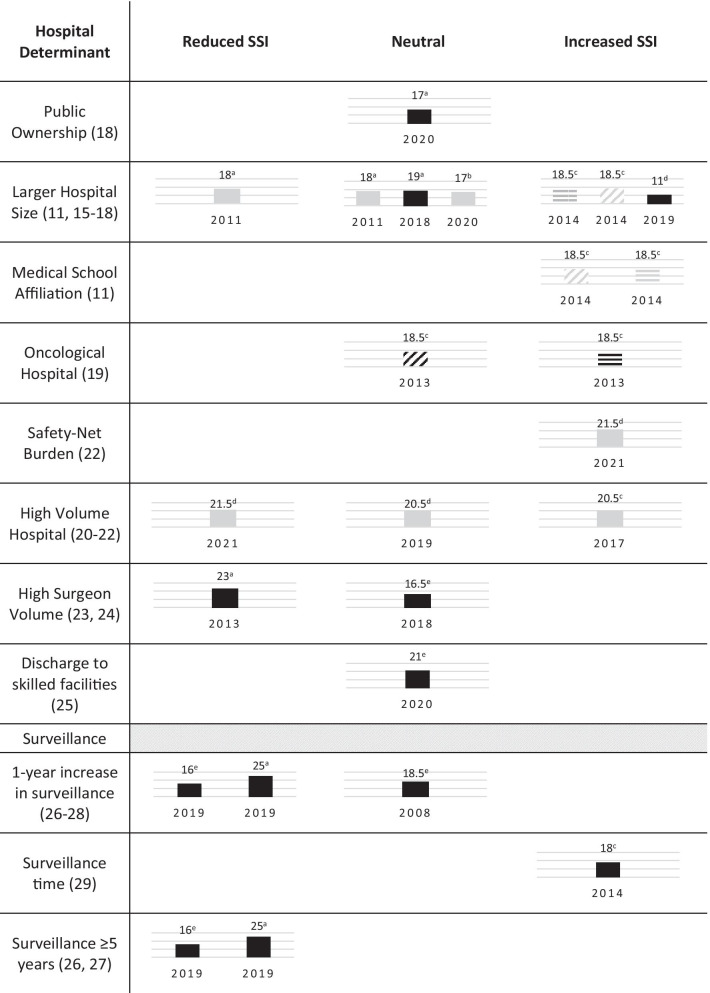
Main findings of included studies, by hospital determinant. Each column refers to a single study. The number on top of each column is the STROBE classification of the study, and the number below is the year it was published. **a** maximum STROBE score of 29, **b** maximum STROBE score of 24, **c** maximum STROBE score of 28, **d** maximum STROBE score of 30, **e** maximum STROBE score of 27. Black columns refer to adjusted associations, grey refer to crude. Full columns refer to overall SSI as outcome, horizontal strips refer to superficial infection and diagonal strips to deep and organ/space infections

Figure 2 summarizes the main findings per hospital determinant. Six studies [11, 15–19] addressed structural variables—hospital size, ownership, affiliation and being an oncological hospital. Two out of the five evaluating hospital size adjusted their analysis for patient and procedural risk factors, finding that larger hospitals were either associated with higher SSI [16] or had no association [18]. In Germany, ownership type was not associated with SSI following colon procedures [18]. In the U. S., crude estimates suggest that hospitals with medical school affiliation were associated with higher incisional and organ/space SSI [11] compared with hospitals without that affiliation, whilst oncological hospitals in the same country were associated with higher superficial SSI incidence (but not organ/space) compared to non-specialized hospitals, independently of patient demographics, procedural risk factors and surgical complexity [19].

Five studies addressed how hospital or surgeon caseload associates with SSI. Hospital volume was defined as the annual volume of colorectal surgeries performed in hospitals [20], the average annual number of rectal procedures [21] or the colectomy case volume only [22]. All presented crude estimates, each reaching a different conclusion (Fig. 2). One study concluded that less experienced surgeons were associated with more postsurgical complications—SSI and others [23], while in the other no significant difference was found between high and low volume surgeons, though medium volume surgeons had significantly less SSIs than high volume ones [24]. Crude analysis suggests that higher safety-net burden may be associated with increased SSI rates [22], and the study evaluating post-discharge destinations found no difference in SSI rates between patients discharged home versus patients discharged to skilled facilities, after adjusting for 19 endogenous and exogenous risk factors [25].

The impact of surveillance over time on SSI rates was evaluated in four studies. In a large international study from 2019, each additional year of surveillance was associated with a lower SSI frequency, using the former year as reference. Additionally, participating in a surveillance network for over five years was associated with lower SSI rates [26]. The same conclusions were found in Italy, in the same year [27], although in the Netherlands, in 2008, no association was found [28]. Contrarily to these findings, one study using data from the Swiss surveillance system showed that time from the start of surveillance to the operation was significantly associated with higher SSI rates in colorectal surgery [29]. All surveillance analyses were adjusted for patient and procedure variables, and are shown in Table 1; Fig. 2.

## Discussion

Although it has been recognized for over 20 years that hospital characteristics may be associated with SSI [5], as they have been shown with other adverse events [30–32], we found few studies addressing them. SSI rates also showed a wide range in incidence, though most use the CDC criteria, suggesting that case identification, as well as follow-up time, may be markedly different across settings, a major issue to be addressed given that SSI incidence is commonly used as a quality indicator for benchmarking between institutions and countries. Moreover, most data retrieved by this review is based on crude estimates, and needs to be interpreted with caution.

Public or private ownership had no apparent association with SSI after colorectal surgery in the German setting, although public hospitals had significantly less SSI after hip prosthesis following arthrosis [18]. A paper from Switzerland, albeit not providing sufficient data for the estimation of measures of association—and thus failing to meet our inclusion criteria—claimed private hospitals had fewer SSI after colorectal surgery [33]. In Australia, a study reported that private hospitals invest significantly less than public institutions in surveillance resources, emphasizing the possible underreporting of infections in the former setting [34]. While the meaning of private and public hospitals is similar throughout the world, the population served, the types of procedures performed, the structural and processual characteristics of hospitals and the financial incentives may differ considerably between countries, precluding the external validity of these conclusions.

The most analyzed hospital characteristic was hospital size. Only two studies [16, 18] provided adjusted ORs for patient and procedure factors, and both considered different cutoff points than their counterparts, who used 500 beds [11, 15, 17], as proposed by the NHSN risk adjustment methodology [6]. One found no evidence of association using 400 beds as a cutoff, though it did find an association between hospital size and all device-associated and ventilator-associated infection, central venous catheter-bloodstream infection, infection by *Clostridioides difficile* and methicillin-resistant *Staphylococcus aureus* [18]. The other concluded that larger hospitals have significantly higher SSI after colorectal surgery, yet it used 1500 and 2500 beds as cutoffs, so the finding may yield no meaning in most countries of the world [16]. Comparisons among countries are also limited for oncological hospitals. While most countries dispose of specialized hospitals in cancer care, National Cancer Institutes are specific to the U. S., as they have a different payment mechanism than other American hospitals and are exempt from reporting all process-of-care and outcome measures to the Centers for Medicare & Medicaid Services [35]. Previously defined safety-net burden is also U. S.-specific. In this case, associations may also be strongly affected by patient case mix. The authors did find a significant association between high burden and in-hospital mortality and general complications, but, unfortunately, no adjusted analysis was conducted disaggregated at the SSI level [22]. Although no difference was found for SSI, discharge to skilled facilities was associated with higher respiratory morbidity, sepsis and vascular thromboembolism [25]. It has been suggested that most SSIs occur after discharge and, thus, may be affected by post-care variables [36], yet we found no other study addressing them.

The three papers on hospital volume [20–22], defined by specific colorectal procedures, provided crude data only. Furthermore, two used ICD-10 to detect in-patient SSI [20, 21], probably underreporting SSI incidence since administrative data has been shown to have limited accuracy for the detection of SSI [37]. Regarding surgeon caseload, the study failing to find an association acknowledged that the small number case may have been insufficiently robust [24].

The positive impact of surveillance on SSI has been widely documented, although many studies focus on total or non-colorectal procedures [38–40], not accounting for the specificities of each SSI. It is accepted that surveillance may decrease SSI rates through two mechanisms: feedback and/or surveillance effect, similar to the Hawthorne effect [41]. At the same time, an artificial increase in the SSI rate may occur, due to changes in case identification, including better registry of previously unreported infections and active post-discharge case finding [36, 42], and due to changes in case mix over time [43]. This artificiality is well supported by a recent study that found a positive correlation between infection rates and audit quality [33], following the biblical sermon: “seek, and ye shall find” [44]. Two papers found that each one-year increase in surveillance time was associated with reduced SSI after colorectal surgery, while one paper failed to find any association. Both positive effects were marginal [0.93 (95%CI 0.89–0.97) in one study [27] and 0.84 (0.79–0.89) in the best year of the other [26]], and could have limited clinical relevance. Relevantly, the influence of the surveillance effect and better case finding tends to wane over time. Hence, both papers concluding that the impact of surveillance is better noticed after the fifth year of its implementation support the impact of feedback on SSI incidence [26, 27]. Longer time trends may be needed to obtain more accurate results, even if an independent effect may exist by hospitals joining surveillance networks at a later point in time, benefiting from national efforts and overall better practices [26]. As opposed to this, one paper found that the longer the time from surveillance to procedure, the higher the SSI rate after colorectal surgery, as well as after appendectomy and knee arthroplasty [29]. Influencing these disparate findings is the fact that some surveillance networks make it mandatory for hospitals to participate, while others have voluntary participation. In the latter, there may be a selection bias similar to a healthy-worker effect, as hospitals in networks tend to allocate more resources towards surveillance when compared to non-included hospitals [26]. On the other hand, participants in voluntary systems are more interested and have more time available for surveillance, and thus are more likely to produce more accurate data [45].

To the best of our knowledge, no systematic review has been published regarding the association between hospital characteristics and SSI. Our search strategy aimed at maximum sensitivity, focusing on three major databases that retrieved a large volume of initial results. It is unlikely that relevant papers were not retrieved as all included studies were written in English and no additional manuscript was found through backward citation. By focusing on SSI after colorectal surgery, we excluded papers evaluating surveillance on SSI as whole. Using the STROBE statement, we found that most papers failed to address how missing data was handled (14 in 16), and to clarify the study’s design in the title or abstract (13 in 16). While study limitations were almost ubiquitously described, they tended to lack the description of the direction and magnitude of identified biases. Due to the heterogeneity found across studies, even when analyzing the same risk factor, we were unable to quantitatively combine study findings in a meta-analysis. Many relevant healthcare delivery variables were not reviewed as we failed to find any study addressing them—that would be the case of nurse staffing, rurality or whether hospitals had an infection control team. Many hospital factors may be highly correlated, as teaching hospitals tend to be larger, urban and have a higher caseload. Healthcare delivery—and its outcomes—is also dependent on regional and national regulations, incentives and the health literacy of the population. Finally, we addressed colorectal surgery as a whole, because most colorectal surgeons perform both colon and rectal procedures. However, they appear to have different SSI rates and, quite possibly, different risk factors [39], and thus it would be relevant to consider studying them as different entities in the future.

## Conclusions

Although there is a paucity of studies addressing hospital-level factors on SSI, surgeon experience and the implementation of surveillance appear to be associated with better outcomes. In order for hospitals and services to be efficiently optimized, more studies addressing these variables are needed that take into account the confounding effect of patient case mix.

## Data Availability

All data generated or analyzed during this study are included in this published article.

## Abbreviations

CDC: Centers for Disease Control and Prevention
CI: Confidence Interval
HAI: Healthcare-Associated Infection
ICD10: International Statistical Classification of Diseases and Related Health Problems, 10th Revision
NHSN: National Healthcare Safety Network
OR: Odds Ratio
RR: Relative risk
SSI: Surgical site infection
STROBE: Strengthening the Reporting of Observational Studies in Epidemiology.
U. S.: United States.
